# Epilepsy Surgery in Kazakhstan: Outcomes and the Role of Advanced Imaging

**DOI:** 10.3390/jcm14227932

**Published:** 2025-11-08

**Authors:** Dina Kalinina, Nazira Bekenova, Alimzhan Muxunov, Zhassulan Utebekov, Gaziz Kyrgyzbay, Darkhan Kimadiev, Guldana Zhumabaeva, Antonio Sarria-Santamera

**Affiliations:** 1 Department of Medicine, School of Medicine, Nazarbayev University, Astana Z05P3Y4, Kazakhstan; alimzhan.muxunov@nu.edu.kz (A.M.);; 2 Epileptology Centre, RSE Medical Centre Hospital of the President’s Affairs Administration of the Republic of Kazakhstan, Astana Z05M4E8, Kazakhstan; nazira.bekenova@mail.ru (N.B.); jase8522@gmail.com (Z.U.); doctorgaziz@gmail.com (G.K.); kimadiev96@gmail.com (D.K.); dana922012@gmail.com (G.Z.)

**Keywords:** drug-resistant epilepsy, epilepsy surgery, Engel outcome, Central Asia, Kazakhstan

## Abstract

**Background and Objectives:** Evidence on epilepsy surgery from Central Asia is limited, reflecting the real-world challenges of developing this service in low- and middle-income settings. We evaluated one-year seizure outcomes after resective surgery for drug-resistant focal epilepsy at a single center in Kazakhstan, and we assessed whether the use of advanced presurgical imaging was associated with seizure freedom. **Materials and Methods:** A retrospective cohort study was conducted, including consecutive adults who underwent curative-intent resective epilepsy surgery from 2017 to 2023. Outcomes at 12 months or more post-surgery were classified using the Engel criteria. Logistic regression was used to examine associations between the advanced presurgical diagnostic tool and achieving an Engel class I outcome. Crude and adjusted risk ratios (RRs) for not achieving Engel I were estimated using modified Poisson regression with robust SEs. **Results:** Among 112 patients (median age 31 years; median epilepsy duration 19 years), 76% underwent temporal lobe procedures and 71% had lobectomies. At one year, 74 patients were seizure-free (Engel II: 15.2%, III: 11.6%, IV: 7.1%). Year-to-year Engel I rates varied without a significant linear trend from 2018 to 2023. In bivariable analyses, MRI-defined atrophy (RR, 3.14) and mixed lesions (RR, 2.62) were associated with a higher risk of not achieving Engel I, whereas longer epilepsy duration was linked to a lower risk (RR, 0.97 per year). In adjusted models, predictors of not achieving Engel I included generalized tonic–clonic seizures (aRR, 1.96), atrophy (aRR, 2.98), mixed lesions (aRR, 2.45), and undergoing any advanced diagnostic test (aRR, 3.38). Longer epilepsy durations remained protective (aRR 0.95 per year). In modality-specific logistic models, fMRI use was associated with higher odds of Engel I (aOR 3.39), and MR spectroscopy was associated with lower odds (aOR 0.33). **Conclusions:** In this Central Asian single-center cohort, about two-thirds of adults achieved complete seizure freedom one year after resective surgery—comparable to international benchmarks. Advanced imaging modalities showed divergent associations with outcomes, likely reflecting confounding by indication. These findings support the feasibility of effective epilepsy surgery in a low-resource context and the value of targeted use of advanced imaging.

## 1. Introduction

Drug-resistant epilepsy (DRE), defined as failure to control seizures with two adequate antiepileptic drug trials, affects roughly 30% of people with epilepsy [1,2,3]. These persistent seizures carry substantial morbidity and highlight the need for alternative treatments [4,5,6]. Epilepsy surgery has emerged as a key therapy for focal DRE, offering a potential cure or significant improvement when seizures originate from a resectable brain region [7,8,9]. In most cases, surgical resection can achieve long-term seizure freedom in appropriately selected patients, with approximately 50–70% of patients in many series attaining Engel class I outcomes [10,11]. Indeed, randomized trials and meta-analyses have confirmed markedly better seizure freedom rates with surgery than with continued medical management [7,8,9]. These data from high-income settings underscore the potential benefit of surgery for DRE and highlight the importance of evaluating outcomes in new programs against these benchmarks.

Accurate localization of the epileptogenic zone before resection is a critical factor in determining surgical success [12,13]. High-resolution structural magnetic resonance imaging (MRI) and video electroencephalogram (EEG) monitoring form the backbone of presurgical evaluation, often identifying lesions (such as hippocampal sclerosis or focal cortical dysplasia) or EEG seizure foci that guide surgical planning [12,14]. However, additional functional imaging modalities can play a valuable role in a subset of patients, particularly those with normal MRI scans or discordant findings [15]. For example, fluorodeoxyglucose-positron emission tomography (FDG-PET) can reveal regional hypometabolism indicative of an epileptogenic focus, and patients with a focal PET abnormality have higher odds of achieving a good postoperative outcome [16,17]. Ictal SPECT scans can similarly highlight seizure onset zones through changes in perfusion [18,19,20]. Advanced MRI-based techniques are also increasingly utilized: functional MRI (fMRI) can map critical functions and delineate epileptic networks in research settings, whereas proton magnetic resonance spectroscopy (MRS) can detect metabolic markers (for example, reduced N-acetylaspartate) in epileptogenic tissue [15,21,22,23,24]. These tools are intended to improve patient selection and outcome prediction [12,15]. For instance, one systematic review noted that novel fMRI methods (such as resting-state fMRI or EEG-fMRI fusion) show promise in enhancing localization for MRI-negative cases, potentially increasing surgical success rates [25]. Likewise, the combined use of PET and MRI has enabled some patients with DRE who are MRI negative to achieve postoperative seizure freedom at rates approaching those of MRI-positive patients [26,27,28]. Thus, the integration of advanced neuroimaging in presurgical workups is increasingly recognized as a means to optimize outcomes, although access to these modalities varies widely by center [29,30].

Epilepsy surgery literature is dominated by reports from North America, Europe, and select centers in Asia, with relatively limited data from Central Asia [31,32,33]. Epilepsy surgery services in low- and middle-income regions frequently face challenges, including limited expertise, funding, and access to technology [30,34,35,36], and advanced presurgical investigations (for example, PET, SPECT, fMRI) are often unavailable outside major urban centers [37,38,39]. Kazakhstan, the largest country in Central Asia, has only recently established comprehensive epilepsy surgery services, and published data remain scarce on the local epilepsy patient population, including its epidemiology and access to care in this setting [31,40,41]. This knowledge gap limits the ability to assess regional practices relative to global benchmarks. In this context, reporting real-world clinical experience from a Kazakhstani center offers relevant insight into the delivery and impact of surgical care in an emerging epilepsy surgery service.

Given the global efficacy of epilepsy surgery and the challenges faced in low-resource environments, there is a need to understand how a developing service in Central Asia performs in practice. This study examined the real-world experience of a single-center cohort of patients with drug-resistant focal epilepsy who underwent resective surgery in Kazakhstan. The primary aim was to describe postoperative seizure outcomes in this cohort at one year, using Engel classification, and to evaluate whether the use of advanced diagnostic imaging tools (such as PET, fMRI, and MRS) was associated with achieving Engel I outcome. By documenting our center’s clinical experience, we also aim to highlight the role and impact of presurgical imaging modalities in an emerging epilepsy surgery service and to contribute data from a region where outcomes have been underreported to date.

## 2. Materials and Methods

### 2.1. Study Design and Setting

A retrospective, single-center cohort of consecutive patients with drug-resistant focal epilepsy who underwent curative-intent resective surgery between 2017 and 2023 at the Presidential Hospital in Astana, Kazakhstan, was performed. The epilepsy surgery program at this hospital (the Epileptology Centre) is one of only four centers in the country that perform epilepsy surgery. It is staffed by a multidisciplinary team, including neurosurgeons and neurologists specializing in epilepsy, as well as support from neuroradiology and neurophysiology. As a national referral center, it evaluates patients from across Kazakhstan. The majority of cases have been adults with focal epilepsy, most often temporal lobe epilepsies associated with identifiable lesions such as mesial temporal sclerosis or low-grade tumors, although extratemporal cases are also managed. This study includes all available cases that meet the eligibility criteria during the specified interval.

The analysis used a de-identified registry approved for research by the institutional review board. This study was conducted under a broader protocol for secondary analysis of de-identified health data, with ethical approval obtained from the Local Bioethics Committee of the Hospital, as documented in Protocol No. 4, dated 20 December 2024, and in accordance with the Declaration of Helsinki. 

### 2.2. Eligibility Criteria

The inclusion criteria were as follows: (i) age ≥18 years at surgery; (ii) drug-resistant epilepsy (failure of ≥2 adequately tried and tolerated antiseizure medications); (iii) resective surgery (temporal lobectomy, lesionectomy, tumor resection); and (iv) documented postoperative outcome at 12 months. The exclusion criteria were as follows: palliative/non-resective procedures (for example, callosotomy, VNS/DBS only), incomplete records preventing outcome classification, and follow-up <12 months.

### 2.3. Presurgical Evaluation and Data Collection

Clinical data were obtained from the epilepsy surgery database and chart review of the hospital. Patient demographics (age at surgery and sex) and clinical history, including epilepsy duration (in years) and seizure characteristics, were collected. Seizure type was classified by semiology and electroclinical data, noting whether patients had focal seizures only or seizures with secondary generalization; we also recorded history of febrile seizures in childhood and episodes of status epilepticus, as these factors were of interest for prognosis. The number and names of antiseizure medications administered before surgery were recorded. We have noted any prior epilepsy surgeries. As part of the presurgical workup, all patients underwent long-term video-EEG monitoring and brain MRI (at 1.5 T or 3 T) to identify potential epileptogenic lesions. No patient underwent invasive intracranial EEG monitoring, as this modality was not available at our center during the study period; all surgical decisions were based on noninvasive data. We documented the presence and type of any lesion on MRI (mesial temporal sclerosis, cortical dysplasia, tumor, vascular malformation) and whether the MRI lesion was concordant with the EEG seizure focus. Additionally, we recorded the use of advanced diagnostic modalities in each patient’s evaluation. Advanced imaging was defined as the use of one or more of the following techniques: interictal FDG-PET, fMRI, proton MR spectroscopy, and diffusion tensor imaging (DTI) tractography. These tests were used selectively to aid localization, map eloquent cortex, or support surgical planning in cases with anatomical or functional complexity. For each modality, we documented whether it was performed during the presurgical workup. The surgical details, including the type of resection (temporal lobectomy, lesionectomy, or tumor resection) and the side of surgery (left or right hemisphere), were obtained from operative reports. Histopathological examination of resected tissue confirmed concordance between pathological, clinical, and imaging-based presurgical diagnoses in all cases. The postoperative seizure outcome was assessed by the treating epileptologist at regular follow-up visits after 12 months. For consistency, the Engel Epilepsy Surgery Outcome Scale was used to classify outcomes at the last available follow-up (at least 1 year post-surgery for all patients). Engel class I was defined as completely seizure-free or only occasional auras since surgery, class II as rare, class III as worthwhile improvement (>50% seizure reduction), and class IV as no meaningful improvement. For certain analyses, we dichotomized outcomes into “seizure free” (Engel I) versus “not seizure free” (Engel II–IV).

### 2.4. Variables

The prespecified covariates were age at surgery, sex, epilepsy duration (in years), febrile seizure history, presence of focal auras, history of generalized tonic–clonic seizures, prior epilepsy surgery, MRI lesion category, EEG–MRI concordance, surgery type, and side. Advanced imaging indicators (PET, fMRI, MRS, and DTI) were coded as binary (performed or not).

### 2.5. Statistical Analysis

Cohort characteristics were summarized as mean (SD) or median (IQR) for continuous variables and counts (percentages) for categorical variables. Outcomes were dichotomized as Engel I versus Engel II–IV for all comparative models. For comparisons of baseline patient characteristics by seizure outcome or surgery site, *p*-values were calculated using Fisher’s exact test for categorical variables, the Wilcoxon rank-sum test for non-normally distributed continuous variables, and t-tests for normally distributed variables, as appropriate. Temporal patterns in seizure freedom were evaluated by modeling the annual Engel I proportion using a binomial framework with the calendar year as a continuous predictor. Operationally, yearly counts were analyzed with a Poisson log-linear model, including the log of the yearly total as an offset. The year 2017 was excluded (program start; *n* = 2). We reported the estimated annual percentage change (APC) with 95% CIs and a two-sided *p*-value for linear trend over 2018–2023. Associations between advanced presurgical diagnostic tests and Engel I outcome were assessed with univariable logistic regression for each modality and a multivariable logistic model including all four modalities; results are shown as odds ratios (ORs) with 95% CIs. Crude and adjusted risk ratios (RRs) for not achieving Engel I at 12 months were estimated using modified Poisson regression with a log link and robust standard errors: crude RRs came from single-predictor models, and adjusted RRs from a multivariable model entering all prespecified predictors (demographic, clinical, imaging, and surgical variables) simultaneously. A two-sided *p* < 0.05 was considered statistically significant. Analyses were performed in R version 4.3.0.

## 3. Results

### 3.1. Patient Characteristics

As shown in Table 1, of the 112 patients who underwent resective epilepsy surgery, 74 (66%) achieved seizure freedom (Engel class I) while 38 (34%) did not (Engel classes II–IV). The cohort comprised 58% males overall, with a similar male proportion among patients who became seizure-free (61%) and those who did not (53%). The median age at surgery was 31 years (interquartile range [IQR] 27–36) in the cohort, with comparable medians in the seizure-free and non-seizure-free groups (30 and 31 years, respectively). The mean duration of epilepsy was 19.7 years (SD 9.9); patients who achieved Engel I had a slightly longer mean disease duration (21.2 ± 10.6 years) than those with persistent seizures (16.7 ± 7.7 years). The median preoperative seizure frequency was 4.5 seizures per month (IQR, 3–10) overall; median frequencies were 6 seizures per month in the seizure-free group and 4 seizures per month in the group that was not seizure-free. A history of febrile seizures (present in 29% of patients overall), the presence of auras (57%), and a history of generalized tonic–clonic seizures (15%) were all similar between outcome groups, with no notable differences between those who did and did not achieve Engel I outcome.

MRI findings and surgical details were similarly distributed across outcomes in most categories. Hippocampal sclerosis was the most common MRI lesion (46% of patients) and was somewhat more frequent in those who became seizure-free (51%) than in those who did not (34%). The prevalence of tumors (18% overall) and focal cortical dysplasia (13%) was similar between seizure outcome groups. In contrast, MRI evidence of cerebral atrophy was more often observed among patients who failed to become seizure-free (11% compared with 1%). Prior epilepsy surgery was rare (9% of patients) and occurred at similar rates in both outcome groups. Regarding the surgical procedure, lobectomy was the most common surgery performed (71% of all cases) and was more frequently performed in patients who achieved Engel I (77% of seizure-free patients and 61% of those not seizure-free). Conversely, lesionectomy accounted for 17% of procedures overall and was relatively more common among patients who did not achieve Engel I outcome (24% compared with 14%). Surgical laterality was evenly split (50% of resections were left-sided overall) with no meaningful difference by outcome. The majority of resections involved the temporal lobe (76% of surgeries); temporal lobe surgeries were more common in those who became seizure-free (80% and 68%), whereas extratemporal surgeries were more common in those with ongoing seizures (32% and 20%).

Of all baseline characteristics compared between patients with Engel I outcome and those with Engel II–IV outcomes, only epilepsy duration differed significantly between groups (*p* = 0.011), with longer duration among those who achieved seizure freedom. All other clinical, imaging, and surgical variables showed no statistically significant differences.

Subgroup comparison by surgery type (temporal and extratemporal) revealed significant differences in lesion type, surgery type, aura history, and febrile seizures (Supplementary Table S1). Hippocampal sclerosis was predominant among temporal cases, while focal cortical dysplasia was more common in extratemporal resections.

### 3.2. Temporal Trend in Engel I Outcome (2018–2023)

**Figure 1:**
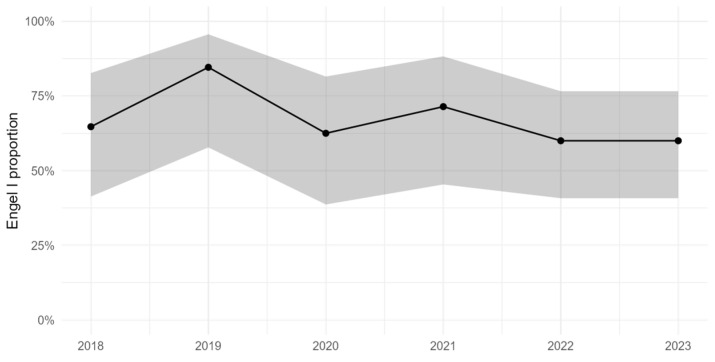
Yearly Engel I rate with 95% CI (2018–2023).

Figure 1 illustrates the annual seizure-free rate (Engel class I) from 2017 through 2023 with 95% confidence intervals. Year-to-year Engel I outcome rates fluctuated over this period, peaking around 2019 and then stabilizing at approximately 60% in 2022–2023. The confidence intervals for these yearly estimates overlapped substantially (Figure 1). The estimated annual percentage change in Engel I rate was −3.56% (95% CI: −15.24 to 9.96; *p* = 0.584), indicating no significant monotonic increase or decrease in Engel I outcome over time. Overall, the proportion of patients achieving Engel I outcomes remained essentially stable across the study period, varying only within the limits of sampling variability.

### 3.3. Advanced Diagnostic Modalities and Surgical Outcome

Use of advanced presurgical diagnostic modalities was common in this cohort. A total of 86 out of 112 patients (76.8%) underwent at least one advanced diagnostic test (such as PET, fMRI, MR spectroscopy, or DTI), as shown in Table 2. The Engel I outcome was achieved in 60.5% (52/86) of patients who underwent any advanced diagnostic testing, compared to 84.6% (22/26) of those who did not undergo any advanced modality. Outcome differences by specific modality were variable (Table 2). The proportion of patients achieving Engel I was approximately the same for those with or without FDG-PET (64.7% compared with 67.2%). Patients who underwent fMRI had a higher seizure-free rate than those who did not (76.2% compared with 53.1%). In contrast, patients who underwent MRS had a lower seizure-free rate compared to those who did not receive MRS (57.4% compared with 76.5%). DTI tractography was performed in only 5 patients; in this small subset, the Engel I rate was 80.0%, compared with 65.4% among patients who did not undergo DTI.

To further evaluate the relationship between advanced diagnostics and surgical outcomes, we examined these variables in logistic regression analyses (Table 3). In univariable logistic models, undergoing any advanced presurgical test was associated with lower odds of achieving Engel I outcome (odds ratio [OR] 0.28, 95% CI 0.08–0.80; *p* = 0.029). Looking at individual modalities, fMRI use was associated with increased odds of Engel I outcome (OR 2.83, 95% CI 1.28–6.45; *p* = 0.011), whereas MR spectroscopy was associated with decreased odds of Engel I outcome (OR 0.41, 95% CI 0.18–0.93; *p* = 0.036). In contrast, use of FDG-PET showed no clear association with outcome (OR 0.89, 95% CI 0.41–1.97; *p* = 0.78). The odds ratio for DTI tractography was elevated but very imprecise due to the small sample (OR 2.11, 95% CI 0.30–42.16; *p* = 0.51).

In a multivariable logistic model adjusting for all four modalities simultaneously (Table 3), the associations with fMRI and MRS persisted. fMRI use remained independently associated with a higher likelihood of Engel I outcome (adjusted OR 3.39, 95% CI 1.46–8.25; *p* = 0.005), whereas MR spectroscopy use remained associated with lower odds of Engel I (adjusted OR 0.33, 95% CI 0.13–0.78; *p* = 0.014). The use of FDG-PET continued to show no significant association after adjustment (adjusted OR 0.73, 95% CI 0.31–1.71; *p* = 0.50). The adjusted OR for DTI tractography was notably high (4.40) but not significant, given the very wide confidence interval (95% CI 0.56–92.8; *p* = 0.20), reflecting the uncertainty from the small number of DTI cases.

### 3.4. Determinants of Engel I Outcome

In the unadjusted (bivariable) analysis of all candidate variables, four factors showed notable associations with seizure outcome. First, patients with MRI-defined cerebral atrophy had a substantially higher risk of not achieving Engel I compared to those with hippocampal sclerosis (RR 3.14, 95% CI 1.65–5.96; *p* = 0.00048). Second, patients with mixed MRI lesions (multiple co-existing lesion types) also had an elevated risk of an unfavorable outcome relative to hippocampal sclerosis (RR 2.62, 95% CI 1.03–6.61; *p* = 0.042). Third, patients who underwent any advanced presurgical diagnostic test had a higher risk of failing to become seizure-free (RR 2.57, 95% CI 1.01–6.57; *p* = 0.049). Fourth, longer duration of epilepsy was associated with a modestly lower risk of not achieving Engel I outcome: each additional year of epilepsy history corresponded to an RR of 0.97 (95% CI 0.95–0.99; *p* = 0.007) for an unfavorable outcome.

None of the other baseline clinical or surgical factors exhibited a significant unadjusted association with seizure outcome (all *p* > 0.05). In particular, there were no clear differences in outcome by patient age or sex, preoperative seizure frequency, history of febrile seizures or auras, or by most MRI lesion etiologies such as tumor, focal cortical dysplasia, residual post-hemorrhagic lesions, or cystic lesions. Surgical variables—including temporal versus extratemporal resection site, right versus left hemisphere, prior epilepsy surgery, and resection type (lesionectomy or tumor resection versus lobectomy)—also showed no notable univariate associations with Engel I outcome. Notably, vascular malformations were present in a very small subset of patients, and none of those patients had recurrent seizures (Engel II–IV outcomes); this resulted in a near-zero risk ratio for vascular lesion cases, an estimate driven by zero events rather than a true protective effect.

**Figure 2:**
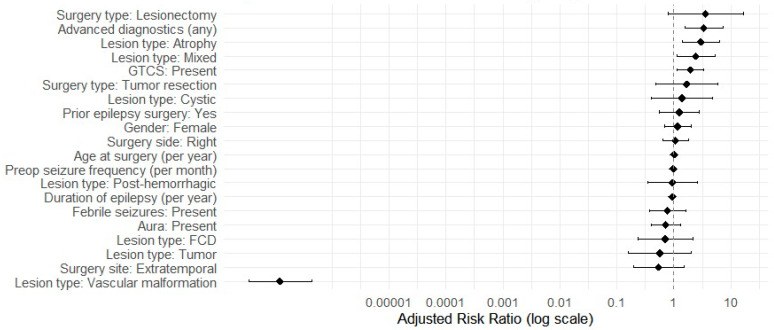
Forest plot of adjusted risk ratios (aRR) for not achieving Engel I at 12 months from a modified Poisson model with robust standard errors. Points show aRRs and horizontal bars the 95% CIs on a log scale; the dashed line marks neutrality (RR = 1). All comparisons are made against the following reference groups: hippocampal sclerosis (HS) for lesion type, lobectomy for surgery type, no advanced diagnostics, left hemisphere, male, no prior epilepsy surgery, no generalized tonic–clonic seizures, no aura, and absence of febrile seizures. GTCS—Generalized Tonic–Clonic Seizures; FCD—Focal Cortical Dysplasia; Preop—Preoperative.

In the adjusted multivariable analysis using a modified Poisson regression with robust variance (Figure 2), several factors emerged as independent predictors of outcome. Patients with preoperative generalized tonic–clonic seizures had nearly twice the risk of not achieving Engel class I compared to those without generalized seizures (adjusted risk ratio [aRR] 1.96, 95% CI 1.14–3.38; *p* = 0.015). MRI-defined atrophy remained strongly associated with a worse outcome: compared to hippocampal sclerosis, atrophy conferred an almost three-fold higher risk of failing to reach Engel I (aRR 2.98, 95% CI 1.41–6.30; *p* = 0.004). Similarly, mixed MRI lesion pathology was linked to a higher risk of persistent seizures relative to hippocampal sclerosis (aRR 2.45, 95% CI 1.12–5.36; *p* = 0.025). Undergoing any advanced diagnostic modality was also independently associated with a higher risk of not being seizure-free (aRR 3.38, 95% CI 1.55–7.38; *p* = 0.002), consistent with the possibility that advanced tests were more often utilized in more complex or refractory cases. By contrast, longer epilepsy duration was associated with a significantly lower risk of an unfavorable outcome in the multivariable model: for each 1-year increase in duration, the risk of failing to achieve Engel I decreased by about 5% (aRR 0.95, 95% CI 0.91–0.98; *p* = 0.003).

No other covariates showed any significant association with seizure outcomes after multivariable adjustment. Variables that were not independently linked to Engel class I outcome included patient age and sex, preoperative seizure frequency, history of febrile seizures or aura, other MRI lesion types (tumor, focal cortical dysplasia, residual post-hemorrhagic changes, or cystic lesions), surgical site (extratemporal compared with temporal lobe), surgery laterality (right or left hemisphere), surgery type (lesionectomy or tumor resection or lobectomy), and prior epilepsy surgery. For all these factors, adjusted risk ratios were near 1.0, and their confidence intervals included unity, with *p*-values exceeding 0.05, indicating no statistically significant influence on the likelihood of an Engel I outcome in this cohort.

### 3.5. Surgical Complications

Among the 112 patients, one postoperative complication was documented: a subarachnoid hemorrhage. The patient recovered without lasting neurological deficit. No other major surgical or anesthetic complications were recorded.

## 4. Discussion

In this retrospective cohort from Kazakhstan, real-world experience with resective epilepsy surgery yielded seizure outcomes comparable to those reported in well-established centers worldwide, despite the constraints of a developing epilepsy surgery service [42,43,44]. Approximately two-thirds of our patients achieved complete seizure freedom (Engel class I) one year after surgery. This rate of Engel I outcome (66%) aligns with the middle of the range reported in the literature for mixed epilepsy surgery populations, where approximately 50–70% of patients become seizure-free depending on the case mix [11,45]. Notably, our cohort comprised patients with MRI-visible lesional epilepsy. Hippocampal sclerosis and low-grade tumors are the most common pathologies, and these etiologies have higher surgical success rates [46]. Consistent with this, subgroup analysis showed that hippocampal sclerosis was far more common in temporal lobe surgeries, while focal cortical dysplasia was more frequent in extratemporal resections [47,48,49]. For example, large meta-analyses have shown that patients with mesial temporal sclerosis or tumor have a roughly 75–80% chance of achieving Engel I outcome, whereas those with nonlesional epilepsy have lower success rates (around 50%) [10,50,51,52]. The favorable Engel I proportion in our series likely reflects this bias toward lesional cases [44,46]. In essence, outcomes are often excellent when a clear epileptogenic lesion is identified and resected; our findings reinforce this principle in the context of a Central Asian surgical center.

One interesting observation in our dataset was the relationship between epilepsy duration and outcome. The cohort’s mean duration was nearly 20 years. In univariable analysis, each additional year of epilepsy was linked to a 3% lower risk of not achieving Engel I (RR 0.97, 95% CI 0.95–0.99; *p* = 0.007). This direction persisted after adjustment: every 1-year increase in duration was associated with a 5% lower risk of failing to achieve Engel I (aRR 0.76, 95% CI 0.63–0.91; *p* = 0.003). In contrast to our cohort’s finding, most studies report that shorter epilepsy duration prior to surgery is associated with higher odds of postoperative seizure freedom [44,53,54]. Several factors could explain the paradoxical trend observed here. One possibility is selection bias coupled with confounding by disease severity: patients with very long-standing epilepsy who ultimately underwent surgery at our center may represent a subset with less aggressive seizures or clearly localizable lesions (for example, long-standing mesial temporal sclerosis), whereas those referred earlier in their disease course likely included individuals with more severe, rapidly progressive epilepsy or diffuse epileptogenic networks that inherently carry a lower probability of cure. This scenario would enrich the long-duration group with surgically remediable cases (biasing outcomes in its favor) while the short-duration group contains more challenging, refractory cases. Additionally, center-specific referral patterns in a developing epilepsy surgery service could contribute—historically, many patients in our setting receive surgery only after exhausting prolonged medical therapy, so the surgical cohort is skewed toward chronic cases that were deemed operable. Overall, larger cohort studies and predictive models have identified longer preoperative epilepsy duration as a risk factor for worse surgical outcomes, in line with meta-analytic evidence that emphasizes the benefits of timely surgical intervention [53]. Thus, the apparent protective effect of a longer duration in our analysis should be interpreted cautiously as an artifact of cohort characteristics and case mix, rather than evidence that delaying surgery improves outcomes.

Our experience also provides insight into the use of advanced presurgical imaging in a resource-limited setting. We observed a moderate uptake of functional imaging, with fMRI utilized in 56% of patients and MRS in 55%, in addition to routine MRI and EEG for all patients. Interestingly, the use of fMRI and MRS showed opposing associations with outcomes. In our adjusted analysis, patients who underwent fMRI had higher odds of achieving Engel I outcome, whereas those who underwent MRS had lower odds. The observed association between fMRI use and seizure freedom likely reflects confounding by indication rather than a causal effect. At our center, fMRI was used selectively for functional mapping—mainly language localization—in patients with dominant-hemisphere lesions, often with clear hippocampal sclerosis. These patients already had favorable prognostic features, including well-localized epileptogenic zones, and were strong surgical candidates irrespective of fMRI use. Thus, the correlation with Engel I outcome likely reflects underlying case characteristics rather than any direct effect of fMRI. This is consistent with prior studies, which emphasize that fMRI’s role is to guide resection strategy and minimize postoperative deficits, not to influence seizure outcome [15,55,56,57]. Major consensus statements and reviews similarly conclude that presurgical fMRI is most useful for noninvasive mapping of eloquent cortex, especially language, and serves as an adjunct to surgical planning in selected cases with dominant hemisphere involvement [58,59,60]. The finding in our study should be interpreted accordingly—as a marker of lower-risk, lesional epilepsy undergoing precautionary mapping—rather than an independent predictor of outcome. MRS, in contrast, is generally reserved for cases where localization remains unclear after conventional evaluation [61,62,63]. Its use in our cohort likely marked patients with more ambiguous MRI findings—cases inherently associated with lower surgical success rates. The inverse association between MRS use and seizure freedom should not be interpreted as a detrimental effect of MRS itself, but rather as another example of confounding by indication: patients referred for MRS likely represented a more diagnostically complex and prognostically challenging group. This interpretation aligns with existing literature, which emphasizes the adjunctive value of MRS in selected cases while recognizing its limited role when core diagnostic tools already provide clear localization [64,65,66].

Our findings suggest that the use of advanced tests often reflects case complexity rather than influencing better or worse outcomes. Consistent with contemporary guidance, a tiered presurgical strategy is recommended: PET, SPECT, fMRI, and MRS are deployed when standard MRI and scalp EEG do not yield a clear target, whereas concordant MRI–EEG cases can proceed without multiple adjunctive studies [12,15,42,58,59,60]. In our center, advanced imaging was largely reserved for patients needing additional localization, which helps explain why those receiving “any advanced diagnostics” showed lower unadjusted Engel I rates. When positive, advanced imaging modalities such as FDG-PET and MRS can support seizure control prediction, particularly in temporal lobe epilepsy. FDG-PET is especially useful in MRI-negative cases, where ipsilateral hypometabolism concordant with EEG findings offers strong lateralizing value and has been associated with Engel I outcomes in 60–70% of well-selected patients [16,17,55,67,68,69,70,71]. In contrast, SPECT—particularly ictal SPECT—has shown more variable utility depending on timing and technical expertise, but it may provide additive value in complex or non-lesional cases [15,19,72,73]. In our cohort, SPECT was not performed due to local unavailability, and PET was selectively used, primarily in cases where standard MRI and EEG data were inconclusive. Compared with published series from high-resource centers that integrate PET or SPECT routinely in surgical planning, our outcomes were still comparable. This may reflect the predominance of lesional, MRI-positive cases in our cohort and the prioritization of patients with high-confidence localization based on noninvasive studies. Nonetheless, broader access to functional imaging could enable more confident surgical planning in cases with subtle or discordant findings, and potentially increase the pool of eligible candidates. We did not analyze test results (only whether a test was performed), so patients undergoing MRS might have included both those with helpful focal findings and those with normal studies or non-lesional evaluations. Overall, advanced imaging remains an important adjunct when used selectively, after maximizing the yield of core evaluations.

Comparing our findings with those of other regions, the results are encouraging and illustrative of how patient selection shapes outcome statistics. Established epilepsy surgery centers often report Engel I outcome in approximately 60–80% of patients, with higher rates in temporal lobe epilepsy and lower rates in extratemporal or nonlesional cases [10,74,75]. For example, a systematic review noted seizure-free rates of approximately 73% for temporal lobe resections compared with 60% for extratemporal resections [76]. Our overall Engel I rate of 66% agrees with these figures, given that our series included a mix of temporal and extratemporal surgeries (though the majority were temporal lobe cases with lesions). A recent report from a neighboring region is worth highlighting: Armenia’s first epilepsy surgery program achieved an impressive 89% Engel I outcome in a highly selected cohort of 28 patients [77]. All patients had lesional epilepsies, and many underwent PET imaging abroad. The Armenian experience demonstrates how strong international collaboration and careful case selection (focusing initially on the most favorable candidates) can yield excellent outcomes in a new program. Our center’s experience is comparable, as we also prioritized clearly identifiable cases. Although our Engel I rate did not reach 90%, it falls within expected ranges, confirming that a competent surgical team with basic diagnostic resources can achieve outcomes on par with international benchmarks. We anticipate that our outcome metrics may evolve as we incrementally include more complex patients, for example, those with inconclusive imaging who will require advanced evaluations. Ongoing comparison with global data is important to ensure that our results remain competitive and to identify any gaps in care.

This study has several limitations that should be acknowledged. First, the retrospective design and single-center scope impose inherent biases. Data accuracy (for example, seizure frequency or Engel classification) depends on clinical records and patient self-report, which can be imperfect. Selection bias is a prominent concern: as discussed, our cohort largely consisted of patients with readily localizable, lesional epilepsy, since we felt confident to operate on, given the resources at hand. Patients with ambiguous findings or widespread epilepsy were less likely to undergo surgery during the study period. This means that our results likely overestimate Engel I outcome rates compared with an unselected DRE population. We have reported on the “easier” surgical cases. Second, the sample size (112 patients) provided limited power for subgroup analyses. Access to advanced diagnostics and surgical adjuncts was uneven, leading to heterogeneity in presurgical evaluation and possible selection of more complex, lower-yield cases for advanced testing. Third, our follow-up duration was relatively short-term (with most patients’ outcomes measured at one year). While one-year Engel outcomes are a standard benchmark, longer-term follow-up is necessary to determine if the Engel I outcome is sustained or if relapses occur after several years. Prior studies have shown that initial postoperative remission can sometimes be followed by late recurrence of seizures [44,78,79]. Moreover, the generalizability of our findings may be limited by the single-center design and country-specific context. Differences in referral pathways, diagnostic capacity, and patient profiles across centers mean that our results should be interpreted with caution outside similar clinical settings.

Despite these limitations, our study provides useful guidance for presurgical evaluation in epilepsy centers and may be informative for other institutions in similar low- or middle-income settings seeking to develop or expand surgical services. The experience in Kazakhstan suggests that, even with selective access to advanced diagnostics for some patients, an epilepsy surgery service can be delivered successfully with careful patient selection and multidisciplinary expertise. The key to this success lies in utilizing available technologies—high-quality MRI, thorough EEG monitoring, and clinical evaluation—to identify optimal candidates. By achieving good outcomes in these patients, a center builds confidence and justifies further investment in the service. We also stress the value of gradually expanding diagnostic capabilities: adding PET, MRS, or other advanced neuroimaging can enable the evaluation of more challenging cases (for example, MRI-negative or multifocal epilepsy). Better access to these modalities, along with training in their interpretation, could increase the proportion of patients who can be offered potentially curative surgery and help narrow the treatment gap for DRE in our region. 

## 5. Conclusions

In conclusion, our findings indicate that epilepsy surgery in Kazakhstan can achieve seizure-freedom rates comparable to those reported internationally for appropriately selected patients. Advanced diagnostic tools are ideal for comprehensive care but can be used pragmatically in cases where standard evaluations are insufficient. As centers like ours continue to evolve, balancing resource constraints with the adoption of new technologies will be crucial to enhancing surgical care and expanding its benefits to more patients. Sharing outcomes from Central Asian and other resource-limited contexts is an important step toward optimizing global epilepsy care and ensuring access to effective surgical treatment.

## Figures and Tables

**Table 1:** Clinical characteristics of the cohort overall and by outcome (Seizure-free and Not seizure-free).

	Overall (*n*= 112) ^1^	Engel I (*n* = 74) ^1^	Engel II–IV (*n* = 38) ^1^	*p*-Value ^2^
Gender				0.426
Female	47 (42%)	29 (39%)	18 (47%)	
Male	65 (58%)	45 (61%)	20 (53%)	
Age at surgery (years)	31 (27–36)	30.0 (26–38)	31.0 (28–33)	0.607
Duration of epilepsy (years)	19.7 (9.9)	21.2 (10.6)	16.7 (7.7)	0.011
Preoperative seizure frequency (per month)	4.5 (3.0–10.0)	6 (3–12)	4 (3–8)	0.431
Febrile seizures	32 (29%)	24 (32%)	8 (21%)	0.271
Aura	64 (57%)	45 (61%)	19 (50%)	0.316
Generalized tonic–clonic seizures	17 (15%)	9 (12%)	8 (21%)	0.268
MRI lesion type				0.157
Hippocampal sclerosis	51 (45.5%)	38 (51.4%)	13 (34.2%)	
Tumor	20 (17.9%)	12 (16.2%)	8 (21.1%)	
Focal cortical dysplasia	15 (13.4%)	9 (12.2%)	6 (15.8%)	
Residual post-hemorrhagic	8 (7.1%)	5 (6.8%)	3 (7.9%)	
Cystic lesion	6 (5.4%)	4 (5.4%)	2 (5.3%)	
Atrophy	5 (4.5%)	1 (1.4%)	4 (10.5%)	
Vascular malformation	4 (3.6%)	4 (5.4%)	0 (0.0%)	
Mixed	3 (2.7%)	1 (1.4%)	2 (5.3%)	
Prior epilepsy surgery				0.732
No previous episurgery	102 (91%)	68 (92%)	34 (89%)	
Previous episurgery	10 (8.9%)	6 (8.1%)	4 (11%)	
Surgery type				0.185
Lesionectomy	19 (17%)	10 (14%)	9 (24%)	
Lobectomy	80 (71%)	57 (77%)	23 (61%)	
Tumor resection	13 (12%)	7 (9.5%)	6 (16%)	
Surgery side				0.550
Left	56 (50%)	39 (53%)	17 (45%)	
Right	56 (50%)	35 (47%)	21 (55%)	
Surgery site				0.244
Temporal	85 (76%)	59 (80%)	26 (68%)	
Extratemporal	27 (24%)	15 (20%)	12 (32%)	

^1^ *n* (%); Median (IQR); Mean (SD); ^2^ Wilcoxon rank-sum test, Welch’s two-sample *t*-test, Fisher’s exact test and Chi-square test.

**Table 2:** Outcome Proportions of Patients Who Underwent Advanced Diagnostics.

Advanced Diagnostics Tools	No, n (%)	No + Engel I, n (%)	Yes, n (%)	Yes + Engel I, n (%)
Any advanced diagnostics	26 (23.2%)	22 (19.6%)	86 (76.8%)	52 (46.4%)
FDG-PET	61 (54.5%)	41 (36.6%)	51 (45.5%)	33 (29.5%)
fMRI	49 (43.8%)	26 (23.2%)	63 (56.2%)	48 (42.9%)
MR spectroscopy	51 (45.5%)	39 (34.8%)	61 (54.5%)	35 (31.2%)
DTI tractography	107 (95.5%)	70 (62.5%)	5 (4.5%)	4 (3.6%)

FDG-PET—fluorodeoxyglucose positron emission tomography, fMRI—functional magnetic resonance imaging, MR spectroscopy—magnetic resonance spectroscopy, DTI—diffusion tensor imaging.

**Table 3:** Use of Advanced Presurgical Imaging and Association with Engel I Outcome.

	Univariate		Multivariate	
	OR (95% CI)	*p*-Value	OR (95% CI)	*p*-Value
Advanced diagnostics (any)	0.278 (0.076–0.803)	0.029	-	-
PET-CT	0.894 (0.407–1.969)	0.78	0.73 (0.31–1.71)	0.5
fMRI	2.831 (1.276–6.453)	0.011	3.39 (1.46–8.25)	0.005
MR spectroscopy	0.414 (0.177–0.928)	0.036	0.33 (0.13–0.78)	0.014
MRI tractography	2.114 (0.299–42.164)	0.511	4.40 (0.56–92.8)	0.2

## Data Availability

The data presented in this study are available on request from the corresponding author due to ethical reasons.

## Supplementary Materials

The following supporting information can be downloaded at: https://www.mdpi.com/article/10.3390/jcm14227932/s1, Table S1: Overall clinical characteristics of the cohort by surgery site (Temporal and Extratemporal).

## Abbreviations

The following abbreviations are used in this manuscript:

DRE	drug-resistant epilepsy
MRI	magnetic resonance imaging
EEG	electroencephalogram/electroencephalography (video-EEG)
FDG-PET	fluorodeoxyglucose positron emission tomography
PET-CT	positron emission tomography–computed tomography
SPECT	single-photon emission computed tomography
fMRI	functional magnetic resonance imaging
MRS	magnetic resonance spectroscopy
DTI	diffusion tensor imaging
VNS	vagus nerve stimulation
DBS	deep brain stimulation
