# Supplementary material for: Epilepsy Surgery in Kazakhstan: Outcomes and the Role of Advanced Imaging
Source: J Clin Med. 2025 Nov 8;14(22):7932. doi: 10.3390/jcm14227932 (PMC12653455; doi:10.3390/jcm14227932)
Supplement: Supplementary file 1 [file jcm-14-07932-s001.zip › jcm-3937246-supplementary.pdf]

Supplementary Table S1. *Overall clinical characteristics of the cohort by surgery site (Temporal and Extratemporal)*

	**Overall (N = 112)** [1]	**Temporal (N = 85)** [1]	**Extratemporal (N = 27)** [1]	**p-value**[2]
**Gender**				0.656
Female	47 (42%)	37 (43.5%)	10 (37.0%)	
Male	65 (58%)	48 (56.5%)	17 (63.0%)	
**Age at surgery (years)**	31 (27–36)	32.0 (27.0–36.0)	30.0 (25.0–33.0)	0.118
**Duration of epilepsy (years)**	19.7 (9.9)	20.3 (10.2)	17.8 (9.0)	0.219
**Preoperative seizure frequency (per month)**	4.5 (3.0–10.0)	4.0 (3.0–8.0)	8.0 (3.0–30.0)	0.092
**Febrile seizures**	32 (29%)	29 (34.1%)	3 (11.1%)	**0.027**
**Aura**	64 (57%)	55 (64.7%)	9 (33.3%)	**0.007**
**Generalized tonic–clonic seizures**	17 (15%)	12 (14.1%)	5 (18.5%)	0.552
**MRI lesion type**				**<0.001**
Hippocampal sclerosis	51 (45.5%)	49 (57.6%)	2 (7.4%)	
Tumor	20 (17.9%)	14 (16.5%)	6 (22.2%)	
Focal cortical dysplasia	15 (13.4%)	4 (4.7%)	11 (40.7%)	
Residual post-hemorrhagic	8 (7.1%)	5 (5.9%)	3 (11.1%)	
Cystic lesion	6 (5.4%)	4 (4.7%)	2 (7.4%)	
Atrophy	5 (4.5%)	4 (4.7%)	1 (3.7%)	
Vascular malformation	4 (3.6%)	3 (3.5%)	1 (3.7%)	
Mixed	3 (2.7%)	2 (2.4%)	1 (3.7%)	
**Prior epilepsy surgery**	10 (8.9%)	7 (8.2%)	3 (11.1%)	0.702
**Surgery type**				**<0.001**
Lesionectomy	19 (17%)	1 (1.2%)	18 (66.7%)	
Lobectomy	80 (71%)	77 (90.6%)	3 (11.1%)	
Tumor resection	13 (12%)	7 (8.2%)	6 (22.2%)	
**Surgery side**				0.377
Left	56 (50%)	45 (52.9%)	11 (40.7%)	
Right	56 (50%)	40 (47.1%)	16 (59.3%)	
**Seizure outcome**				0.244
Not seizure-free	85 (76%)			
Seizure-free	27 (24%)			

[1] n (%); Median (IQR); Mean (SD)

[2]Wilcoxon rank-sum test, Welch's two-sample t-test, Fisher's exact test and Chi-square test
